# Mitochondrial Genome Features and Phylogenetic Analyses of Four Chrysochroinae Species (Coleoptera: Buprestidae)

**DOI:** 10.3390/biom15111531

**Published:** 2025-10-30

**Authors:** Jieqiong Wang, Yingying Li, Zhonghua Wei, Aimin Shi

**Affiliations:** The Key Laboratory of Southwest China Wildlife Resources Conservation of the Ministry of Education, College of Life Sciences, China West Normal University, Nanchong 637009, China; jqwang0902@126.com (J.W.); yingyingli045@163.com (Y.L.); aiminshi@cwnu.edu.cn (A.S.)

**Keywords:** Buprestidae, Chrysochroinae, mitogenome, phylogenetic analysis

## Abstract

The number of known mitochondrial genomes in Buprestidae is limited, especially in Chrysochroinae, which seriously hinders the phylogenetic study of this family. The mitogenomes of *Capnodis miliaris*, *Lamprodila cupreosplendens*, *Sphenoptera insidiosa* and *Philocteanus rubroaureus* were sequenced, assembled and annotated in this study. The mitogenomes of these four species are typical circular double-stranded DNA molecules, containing 13 protein-coding genes (PCGS), 22 transfer RNA genes (tRNAs), 2 ribosomal RNA genes (rRNAs), and a control region (CR). The total lengths of these four mitogenomes are moderate, ranging from 15,778 bp to 16,230 bp. Additionally, their A + T content ranges from 68.76% to 73.47%, showing positive AT-skew values ranging from 0.098 to 0.181. Relative Synonymous Codon Usage (RSCU) analysis indicated that TTT (Phe), ATT (Ile), TCT (Ser2), and TTA (Leu2) are the most frequently used codons. The gene arrangement of four mitogenomes is consistent with the previously reported Buprestidae mitogenomes. Most of the PCGs use ATN as the start codon, with TAA as the stop codon or an incomplete stop codon T-. Phylogenetic trees were constructed based on the PCGs and rRNAs using both maximum-likelihood and Bayesian inference methods. The phylogenetic results showed that Julodinae, Polycestinae, Buprestinae and Agrilinae are monophyletic groups, and Chrysochroinae is a paraphyletic group. As the number of Buprestidae mitogenomes used for polyogenetic analysis increases, the topology of phylogenetic tree shows differences compared to previous studies.

## 1. Introduction

The family Buprestidae is one of the largest families in the order Coleoptera, comprising 6 subfamilies, 521 genera, and over 15,000 species [1]. Buprestid beetles are herbivorous, have a worldwide distribution, and exhibit significant morphological diversity across species from different subfamilies [2]. Some species are global pests of agriculture and forestry, especially in Agrilinae [3,4,5] and Buprestinae [6,7], while a few species are endangered [8,9,10]. The subfamily Chrysochroinae is divided into 8 tribes, 112 genera, and approximately 3100 species worldwide, with a broad distribution across the six major zoogeographical regions. Species of this subfamily are large in size; adults all feed on plant leaves, while larvae bore into tree trunks or branches, such as the species of *Lamprodila* [11,12] and *Sphenoptera* [13,14].

The subfamily Chrysochroinae, as a key group within the Buprestidae, has long been a focus of research in entomology due to its classification status and phylogenetic relationships [15,16]. While numerous scholars have conducted in-depth studies on its morphological characteristics, phylogenetic studies on subfamily Chrysochroinae remain relatively scarce [17,18]. Morphological characteristics have provided valuable insights into the classification of Buprestidae, but the phylogenetic relationships among higher-level taxa have not yet been fully resolved [17,18,19]. Previous studies have shown that Chrysochroinae and Buprestinae are polyphyletic, and their interrelationships remain uncertain [18]. The classification of the genus *Coomaniella* Bílý, 1974, has been controversial for a long time [18]. Based on morphological cladistic analysis, the antenna structure is consistent with the typical characteristics of Buprestinae [20], providing solid morphological evidence for the traditional classification of this genus.

The mitogenome is a double-stranded circular DNA molecule, which has unique advantages such as maternal inheritance, a rapid evolutionary rate, and a relatively stable structure. It has become an efficient molecular marker for studying the phylogeny of insect [21,22,23,24]. The mitogenome has been widely applied in entomological research, providing crucial molecular evidence for revealing the evolutionary relationships among different insect species [25,26,27,28]. However, research on the mitogenomes of Chrysochroinae is relatively scarce, which greatly limits the understanding of the phylogenetic relationships among various taxa.

In this study, the complete mitogenomes of *Capnodis miliaris* (Klug, 1829), *Lamprodila cupreosplendens* (Kerremans, 1895), *Sphenoptera insidiosa* (Mannerheim, 1852), and *Philocteanus rubroaureus* (De Geer, 1778) were sequenced and annotated. The mitogenomes of the genera *Capnodis*, *Lamprodila*, *Sphenoptera*, and *Philocteanus* are reported here for the first time. The mitogenomes from this study will provide valuable data for the phylogenetic analysis, species identification, and population genetics of the subfamily Chrysochroinae.

## 2. Materials and Methods

### 2.1. Taxon Sampling and DNA Extraction

In this study, specimens of *Capnodis miliaris* were collected on 17 April 2022, from Xiremu Village in the Gaochang District of Turpan City, Xinjiang Uygur Autonomous Region, China. Specimens of *Lamprodila cupreosplendens* were collected on 13 June 2022, from Wanzi tou in Yunwu Town, Guiding County, Guizhou Province, China. Specimens of *Sphenoptera insidiosa* were collected on 8 July 2024, from Shiyu Village in the Liujang National Geological Park, Qinhuangdao City, Hebei Province, China. Specimens of *Philocteanus rubroaureus* were collected on 28 June 2024, in Tuanshan Village, Yongde County, Yunnan Province, China. All specimens are stored at China West Normal University in a −24 °C environment, preserved in 95% ethanol. The leg and thorax tissues were used for DNA extraction using the DNeasy Blood and Tissue Kit (Sangon Biotech (Shanghai) Co., Ltd., China). The experimental procedures were strictly followed in accordance with the kit instructions.

### 2.2. Mitogenome Sequencing, Annotation and Analysis

In this study, high-throughput sequencing was conducted using the Illumina MiSeq platform with paired-end 150 bp. The sequence assembly followed the method proposed by Hahn [29]. The sequencing coverage for *C. miliaris*, *L. cupreosplendens*, *S. insidiosa*, and *P. rubroaureus* reached 295.9 X, 270.2 X, 215.3 X, and 239.2 X, respectively. Low-quality sequence fragments were removed using Trimmomatic (v0.36) software, and the cleaned sequences were then assembled and annotated using Geneious (v11.0.2) software [30]. During data analysis, the relative synonymous codon usage (RSCU) frequency of the mitogenomes was calculated using MEGA (v12) software [31]. Nucleotide diversity (Pi), nonsynonymous substitution rate (Ka), and synonymous substitution rate (Ks) for protein-coding genes (PCGs) were estimated using DnaSP (v5.0) software [32]. These three mitogenome sequences obtained in this study have been submitted to GenBank, with the accession numbers PV330099 (*C. miliaris*), PV330528 (*L. cupreosplendens*), PV391144 (*S. insidiosa*), and PX370042 (*P. rubroaureus*). 

### 2.3. Phylogenetic Aanlysis

To further investigate the phylogenetic position of these four species within the Buprestidae, 43 mitogenomes (Table S1), including 39 previously reported and 4 newly sequenced mitogenomes representing 5 subfamilies, were used for phylogenetic analysis. The Maximum likelihood (ML) and Bayesian inference (BI) methods were used to construct the phylogenetic trees, with *Dryops ernesti* (Gozis, 1866), *Heterocerus parallelus* (Gebler, 1830), *Pyrocoelia rufa* (Olivier, 1886), and *Limonius minutus* (Linnaeus, 1758) as the outgroups. First, PCGs and rRNAs sequences were all aligned using ClustalW (v2.1) software [33]. The sequences were then trimmed using trimAl (v1.2) software [34]. The concatenate sequence function in PhyloSuite (v1.2.2) software was used to concatenate the aligned sequences of PCGs and rRNAs [16,35], and the best-fit model was determined using ModelFinder (v2.5) software [33]. The Best-fit models for the ML and BI analyses are GTR + F + I + G4 and GTR + I + G + F, respectively. The phylogenetic trees were constructed using IQ-tree (v1.6.8) software [36] and MrBayes (v3.2.6) program [33]. The ML analyses were conducted with the following parameters: the number of bootstraps: 50,000; replicates: 1000; and minimum correlation coefficient: 0.9. For Bayesian analysis, two independent Markov chains were set with a total of 2,000,000 generations, with each chain containing 4 parallel Markov chains and a sampling interval of 100 generations. The burn-in period was set to 0.25. The final phylogenetic tree was edited and visualized using Figtree (v1.4.3) software [33].

## 3. Results

### 3.1. Genome Organization and Base Composition

This study performed the complete sequencing and annotation of the mitogenomes of four Chrysochroinae species: *C. miliaris* (No. PV330099), *L. cupreosplendens* (No. PV330528), *S. insidiosa* (No PV391144), and *P*. *rubroaureus* (No. PX370042). Overall, these four newly sequenced mitogenomes share the same composition, consisting of 37 coding genes (13PCGs, 22 tRNAs and 2 rRNAs) along with a control region (A + T-rich region). Further analysis revealed that four PCGs (*nad1*, *nad4*, *nad4L*, and *nad5*), eight tRNAs (*trnC*, *trnF*, *trnH*, *trnL1*, *trnP*, *trnQ*, *trnV*, and *trnY*), and two rRNAs (*rrnL* and *rrnS*) are encoded by the N strand, while the remaining 23 genes (14 tRNAs and 9 PCGs) are encoded by the J strand (Figure S1).

The A + T content of the four complete mitogenomes ranges from 68.76% to 73.47%, showing a positive AT-skews values varying from 0.098 to 0.181 (Table S1). Regarding gene overlap, the mitogenome of *C. miliaris* contains 12 overlapping regions, with a total length of 34 bp; *L. cupreosplendens* has 13 overlapping regions, with a total length of 37 bp; *S. insidiosa* has 20 overlapping regions, with a total length of 96 bp; and *P. rubroaureus* has 14 overlapping regions, with a total length of 67 bp (Table 1). Notably, the longest overlap in all four species is 35 bp (*S. insidiosa*), located between the *cox2* and *trnK* genes. Additionally, there is a 7 bp overlap between the *atp8* and *atp6* genes, as well as between the *nad4* and *nad4L* genes, which aligns with common features of insect mitogenomes. Worth noting is that *P. rubroaureus* has a 128 bp and 83 bp intergenic region between the *trnD* and *atp8* genes, and between the *rrnL* and *trnV* genes, respectively. Which is the longest intergenic region among the four species.

Comparing with the mitogenomes of other species in the family Buprestidae, the four species provided in this study exhibit high conservation in gene arrangement, nucleotide composition, and codon usage patterns. Gene rearrangement was not detected in these four mitogenomes.

### 3.2. Protein-Coding Regions, Codon Usage and Nucleotide Diversity

There are differences in the total lengths of PCGs among the four mitogenome: *C. miliaris* (11,159 bp), *L. cupreosplendens* (11,150 bp), *S. insidiosa* (11,034 bp), and *P. rubroaureus* (11,140 bp), which account for 66.16–71.93% of their respective mitogenome lengths. Among the 13 PCGs, the *atp8* gene is the shortest, ranging from 156 to 177 bp, while the *nad5* gene is the longest, with a length ranging from 1708 to 1720 bp. In terms of initiation codons, most PCGs use ATN (ATA/ATT/ATG/ATC) as the start codon, while the *nad1* and *cox1* gene are unique in using TTG and ACG as the start codon, respectively. Furthermore, the start codons for *cox1* in *C. miliaris* and *L. cupreosplendens* are not detected, potentially indicating an unusual start codon. Termination codon analysis shows that, except for five genes (*cox1*, *cox2*, *cox3*, *nad5*, and *nad4*) that use incomplete termination codons (T-), which are completed by the addition of an A residue at the 3′ end of mRNA, the remaining genes use TAA or TAG as termination codons [37,38].

**Figure 1:**
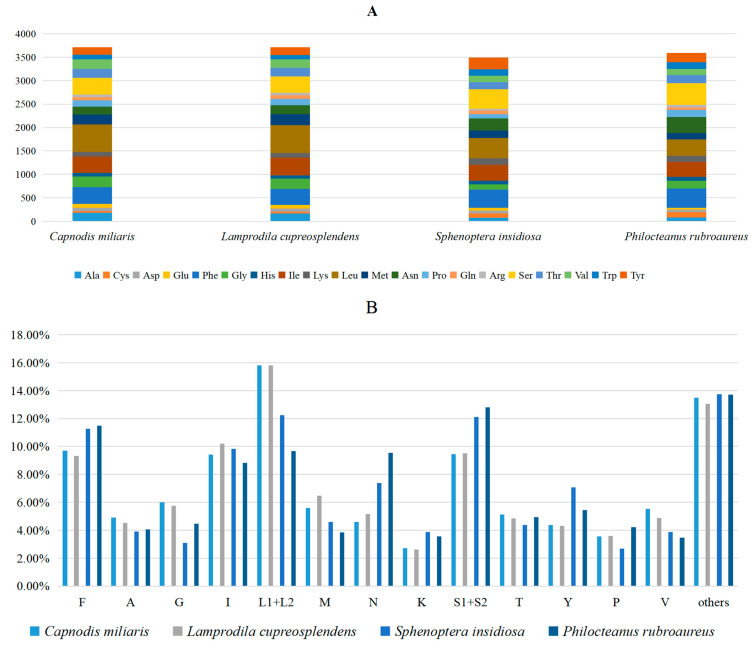
Amino acid composition analysis of four new mitogenome sequences. (**A**) Numbers of different amino acids. (**B**) Percentages of the top twelve amino acids. The stop codon is not included in these graphs.

**Figure 2:**
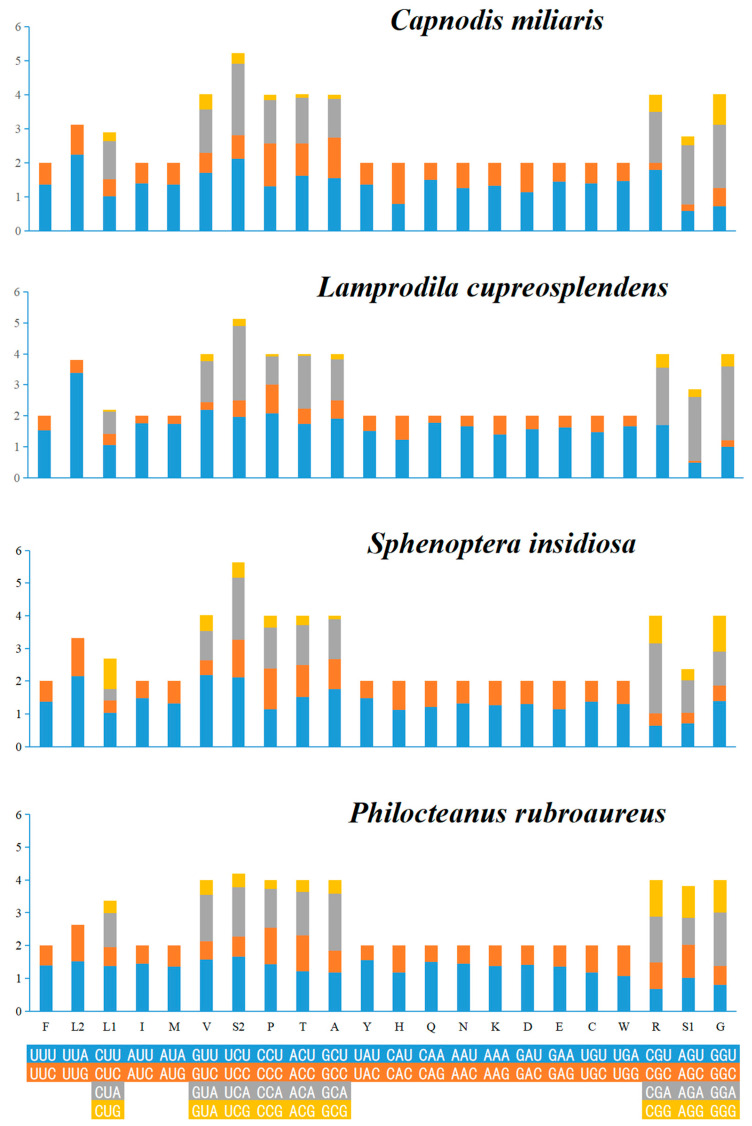
Relative synonymous codon usage of the four new sequenced mitogenome.

The amino acid counts of PCGs in these four mitogenomes (Figure 1) and the relative synonymous codon usage (RSCU) values (Figure 2) are presented. The results indicate that Phe (F), Ile (I), Ser (S2), and Leu (L2) are the four most commonly used amino acids, while the content of other amino acids is below 7.00%. In addition, *C. miliaris* and *L.cupreosplendens* have relatively large total numbers of amino acids, whereas *S. insidiosa* has a relatively small total number. This reflects differences in the length of proteins encoded by the mitogenome. The RSCU values show that TTT (Phe), ATT (Ile), TCT (Ser2), and TTA (Leu2) are the most frequently used codons. The UUU codon for Phe is used significantly more frequently across multiple species, while the UUA and UUG codons of Leu exhibit a strong usage bias. Additionally, the AUU and AUC codons for Ile maintain a high usage frequency. These preferential characteristics are associated with the efficiency of gene expression regulation and the evolutionary adaptability of species, reflecting group-specific codon selection within the mitogenomes of chrysochroine beetles.

**Figure 3:**
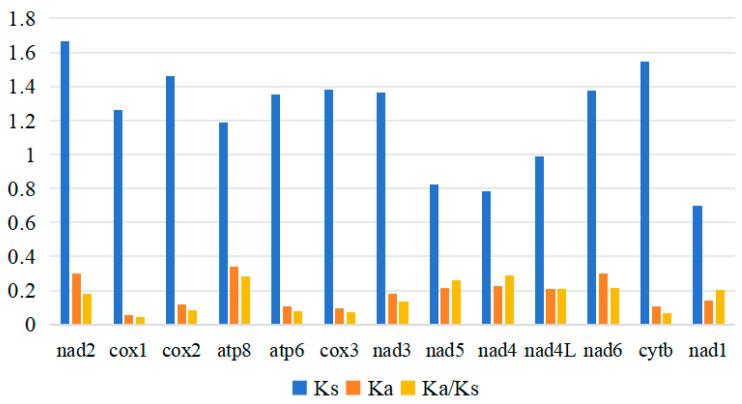
The ratio of Ka/Ks of 13 PCGs in the four newly sequenced buprestid mitogenomes.

The nucleotide diversity (Pi) of PCGs in these four mitogenomes was measured, with results ranging from 0.193 to 0.345. Specifically, the *nad2* (Pi = 0.345) exhibited the highest variability, followed by *nad6* (Pi = 0.337), *atp8* (Pi = 0.33), and *nad3* (Pi = 0.273), while the *cox1* gene showed the lowest variability (Pi = 0.193). By calculating the nonsynonymous substitution rate (Ka), synonymous substitution rate (Ks), and Ka/Ks ratio of PCGs in these four mitogenomes (Figure 3), it was found that the indicators of the *nad4* were significantly higher than those of other genes, suggesting that this gene has a relatively fast evolutionary rate. Among these genes, the *cox1* gene having the lowest value (Ka/Ks = 0.04). This result indicates the presence of purifying selection in these four species [39]. The ATP8 gene has a relatively higher Ka value compared to other genes, and its Ka/Ks ratio is also relatively high. This suggests that the gene experiences weaker purifying selection constraints during evolution; however, it still undergoes purifying selection overall, maintaining the basic stability of mitochondrial functions.

### 3.3. Ribosomal and Transfer RNA Genes

The rRNAs are located in the A + T region between *trnL1* and *trnV*. Both rRNA genes are located on the N strand, with the length of *16S* ranging from 1185 to 1290 bp and the length of *12S* ranging from 607 to 737 bp. Their A + T content is between 72.18% and 76.74%, and the AT-skew values range from −0.16 to −0.03. The total length of the 22 tRNAs is between 1438 and 1452 bp, with individual gene lengths ranging from 61 to 71 bp. The A + T content is between 73.75% and 74.4%, and the AT-skew values range from 0.01 to 0.02. Structural analysis showed that, except for *trnS1*, which cannot form the standard cloverleaf structure due to the absence of the DHU arm, the remaining tRNAs exhibit typical cloverleaf secondary structures. Notably, the *trnS1* genes of the four species are nearly identical in size, measuring 68 bp, 67 bp, 67 bp, and 67 bp (Figures S2–S5). U-G mismatches were also detected in some tRNA genes.

### 3.4. Control Region and Gene Arrangement

The control region (CR), also known as the A + T-rich region or non-coding region, is a core functional unit in the mitogenome. As the longest non-coding region in the mitogenome, it is typically located between *trnI* and *rrnS*. This region exhibits unique evolutionary characteristics, with an evolutionary rate approximately 2.8 to 5 times higher than that of other mitochondrial regions, marked by frequent base substitutions and rich sequence variations.

In this study, the control region lengths of the four species, *C. miliaris*, *L. cupreosplendens*, *S. insidiosa*, and *P. rubroaureus*, were 1581 bp, 1635 bp, 1488 bp, and 1138 bp, respectively. Sequence analysis results showed that the A + T content of the control region (75.66–95.48%) was significantly higher than of other components of the mitogenome, including PCGs (66.16–71.93%), rRNA (72.18–76.74%), and tRNA (70.85–74.4%) (Table 2). Furthermore, compositional analysis indicated that the CR of these four species in subfamily Chrysochroinae exhibited both positive and negative AT-skew values, revealing the diversity of base composition in this region.

### 3.5. Phylogenetic Analysis

**Figure 4:**
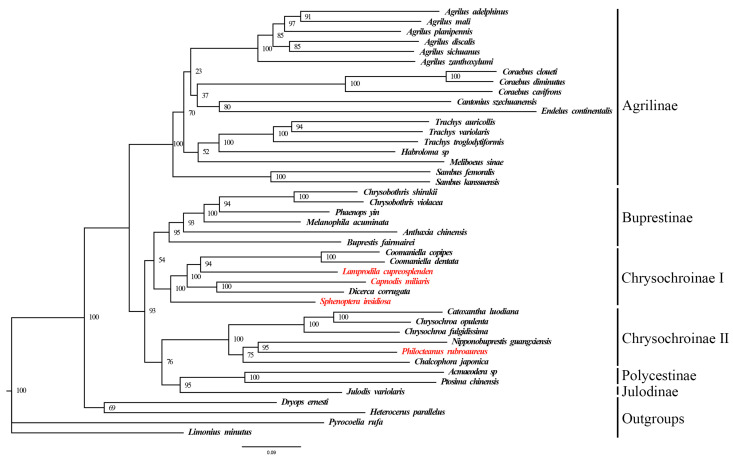
Maximum likelihood tree of 39 Buprestidae based on 13 PCGs + 2 rRNAs. Values at nodes are bootstrap support values. Red names are the target species provided in this study.

**Figure 5:**
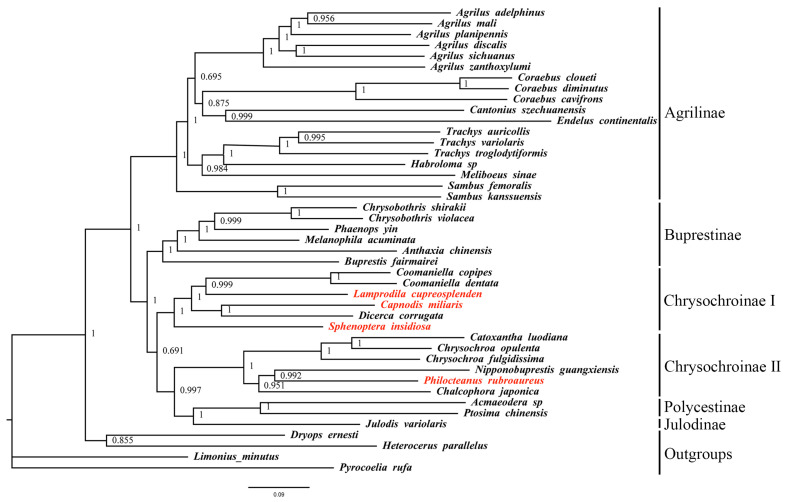
Bayesian tree of 39 Buprestidae based on 13 PCGs + 2 rRNAs. Values at nodes are posterior probability. Red names are the target species provided in this study.

To better understand the phylogenetic position of these four species and the phylogenetic relationships among subfamilies in Buprestidae, this study used a mitogenome dataset (13 PCGs + 2 rRNAs) from 43 species, including four outgroups, to construct ML and BI trees. Despite using two different methods, the topologies of the phylogenetic trees were consistent (Figure 4 and Figure 5). Outgroup taxa and buprestid species were clearly separated, supporting the monophyly of Buprstidae.

Based on the topologies of the ML and BI trees, the Chrysochroniae is a paraphyletic group. The four target species cluster together with other Buprestinae species, forming a clade with low support values. In the Chrysochroinae I clade, ((*Coomaniella copipes* + *Coomaniella dentata*) + *Lamprodila cupreosplendens*) and (*Capnodis miliaris* + *Dicerca corrugata*) are sister groups; in the Chrysochroinae II clade, ((*Catoxantha luodiana* + *Chrysochroa opulenta*) + *Chrysochroa fulgidissima*) and ((*Nipponobuprestis guangxiensis* + *Philocteanus rubroaureus*) + *Chalcophora japonica*) are sister groups.

## 4. Discussion

The mitogenome has many advantages, such as high conservatism, maternal inheritance, and a relatively fast evolutionary rate, making it an important tool in studies of phylogenetic relationships, species identification, and environmental adaptability [21,22,23,24,40]. Additionally, the complete mitogenome not only provides insights into general genomic characteristics but also has the potential to study functional molecular mechanisms [41]. Therefore, in this study, we newly sequenced the mitogenomes of four Chrysochroinae species and combined them with the mitogenome of 39 known buprestid species to construct the phylogenic trees of Buprestidae.

The results indicated that the four newly sequenced mitogenomes contain 37 typical genomic genes and a control region. The gene composition is consistent with the mitogenome characteristics of Buprestidae reported in previous studies [16,42,43,44]. In terms of mitogenome length, it is moderate, mainly influenced by the length of the control region, which is consistent with the previous study [45]. Further analysis revealed the absence of gene rearrangements in these four mitogenomes, which is also consistent with previous studies [42,46]. With respect to base composition, all four new sequences show a distinct AT-skew. The analysis of amino acid usage frequency shows that L2, I, S2, and F are the most frequently occurring amino acids, which corroborates previous studies [42,43,44,45,47]

In the PCGs, the *cox1* gene exhibited the lowest variability (Pi = 0.193), with its Ka/Ks ratio also being the lowest. This suggested that the *cox1* gene had a relatively slow evolutionary rate, consistent with previous studies [16,45]. In tRNAs, except for *trnS1*, all other tRNAs has the typical cloverleaf secondary structure; however, the dihydrouridine (DHU) arm of *trnS1* formed a simple circular structure, differing from the conventional structure [16,44,48,49]. The topologies phylogenetic trees showed significant differences in the phylogenetic relationships between subfamilies compared to previous study [18]. This is mainly attributed to the different genes and different number of inergroup: previous study analyzed four gene fragments from 141 ingroup taxa, whereas this study used mitogenome data from 39 ingroup taxa for phylogenetic analysis.

The results of phylogenetic analysis showed that the subfamily Chrysochroinae is not a monophyletic group, which is divided into two clades. This finding contrasts with previous studies, which indicated that the Coomaniellini and Dicercini tribes were more closely related [45,46,50]. The result of this study may be caused by differences in the number of mitogenomes. It is noteworthy that some researchers support the merger of Chrysochroinae and Buprestinae, suggesting that there is no clear distinction between these two subfamilies [18,51,52,53]. Additionally, in Agrilinae, the classification status of the genus *Sambus* has also been subject to frequent changes [1,42,45,54]. In this study, the relationship between Chrysochroinae and Buprestinae is not well addressed. In the future, mitogenome data from more species of the Buprestidae will be used to explore the phylogenetic relationships among the high-level taxa.

## 5. Conclusions

In this study, the mitogenomes of *C. miliaris*, *L. cupreosplendens*, *S. insidiosa*, and *P.rubroaureus* were sequenced, assembled and annotated. The total lengths of these four mitogenomes ranged from 15,778 bp to 16,230 bp, and their gene arrangement order is completely consistent with that of the known buprestid mitogenomes. These four mitogenomes exhibit the characteristic of high A + T content, which is consistent with the common base composition preference of insect mitogenomes, accompanied by a positive AT-skew and a negative GC-skew. PCGs generally follow the conserved ATN start codon pattern, but specific variations have also been identified: *nad1* in *C. miliaris* and *L. cupreosplendens* starts with TTG, while *cox1* in *S. insidiosa* uses ACG. These exceptions provide valuable insights into the molecular evolutionary characteristics of PCGs. The phylogenetic trees constructed by the two methods have consistent topology, and the results of phylogenetic analysis showed that Chrysochroinae is paraphyletic. The phylogenetic relationship within Chrysochroinae os not well resolved, and it may require more Buprestid mitogenome data or genomic data combing morphological characteristics, to resolve this issue.

## Figures and Tables

**Table 1:** Information of the four newly annotated Chrysochroinae mitogenomes.

Gene	Strand	Position		Codons		Intergenic Nucleotides
		From	To	Start	Stop	
*trnI*	J	1/1/1/1	64/64/64/66			0/0/0/0
*trnQ*	N	77/62/62/64	145/130/130/132			12/−3/−3/−3
*trnM*	J	145/130/130/132	214/198/198/200			−1/−1/−1/−1
*nad2*	J	215/199/253/201	1240/1221/1224/1220	ATT/ATT/ATT/ATA	TAA/TAA/TAA/TAG	0/0/54/0
*trnW*	J	1239/1220/1223/1222	1305/1286/1288/1293			−2/−2/−2/1
*trnC*	N	1298/1279/1281/1286	1363/1340/1341/1346			−8/−8/−8/−8
*trnY*	N	1364/1341/1342/1347	1427/1404/1404/1411			0/0/0/0
*cox1*	J	1429/1406/1403/1404	2959/2936/2941/2944	?/?/ACG/ATT	T/T/TAA/T	1/1/−2/−8
*trnL2*	J	2960/2937/2937/2945	3024/3001/3002/3010			0/0/−5/0
*cox2*	J	3025/3002/3051/3011	3712/3683/3725/3698	ATA/ATA/ATA/ATT	T/T/TAA/T	0/0/48/0
*trnK*	J	3713/3684/3691/3699	3783/3753/3761/3768			0/0/−35/0
*trnD*	J	3783/3754/3761/3769	3847/3818/3826/3832			−1/0/−1/0
*atp8*	J	3848/3819/3827/3961	4003/3974/3985/4137	ATC/ATA/ATT/ATC	TAA/TAA/TAA/TAA	0/0/0/128
*atp6*	J	3997/3968/3979/4131	4671/4642/4653/4805	ATG/ATG/ATG/ATG	TAA/TAA/TAA/TAA	−7/−7/−7/−7
*cox3*	J	4671/4642/4653/4805	5457/5428/5440/5591	ATG/ATG/ATG/ATG	T/T/T/T	−1/−1/−1/−1
*trnG*	J	5458/5429/5440/5592	5521/5490/5503/5654			0/0/−1/0
*nad3*	J	5522/5491/5504/5655	5875/5844/5857/6008	ATT/ATT/ATA/ATA	TAG/TAG/TAA/TAG	0/0/0/0
*trnA*	J	5874/5843/5863/6007	5935/5905/5928/6070			−2/−2/5/−2
*trnR*	J	5935/5905/5928/6071	6001/5968/5994/6132			−1/−1/−1/0
*trnN*	J	6001/5968/5994/6132	6065/6033/6058/6195			−1/−1/−1/−1
*trnS1*	J	6066/6034/6059/6196	6132/6100/6125/6262			0/0/0/−1
*trnE*	J	6133/6101/6126/6265	6195/6162/6190/6326			0/0/0/2
*trnF*	N	6195/6162/6190/6326	6258/6225/6255/6389			−1/−1/−1/−1
*nad5*	N	6259/6226/6255/6399	7978/7945/7967/8106	ATT/ATT/ATT/ATT	T/T/TAA/T	0/0/−1/9
*trnH*	N	7979/7946/7977/8110	8042/8009/8040/8171			0/0/9/3
*nad4*	N	8043/8010/8095/8149	9375/9342/9376/9507	ATG/ATG/ATG/ATG	T/T/T/TAA	0/0/54/−24
*nad4L*	N	9369/9336/9370/9501	9656/9626/9660/9770	ATG/ATG/ATG/ATG	TAA/TAA/TAA/TAA	−7/−7/−7/−7
*trnT*	J	9659/9629/9663/9791	9724/9692/9727/9853			2/2/2/20
*trnP*	N	9725/9693/9727/9854	9789/97,575/9792/9918			0/0/0/0
*nad6*	J	9791/9759/9794/9923	10,294/10,262/10,303/10,423	ATA/ATA/ATT/ATA	TAA/TAA/TAA/TAA	1/1/1/4
*cytb*	J	10,298/10,262/10,303/10,423	11,440/11,404/11,445/11,562	ATG/ATG/ATG/ATG	TAG/TAG/TAG/TAG	3/−1/−1/−1
*trnS2*	J	11,439/11,403/11,444/11,561	11,506/11,470/11,510/11,628			−2/−2/−2/−2
*nad1*	N	11,543/11,489/11,528/11,646	12,487/12,439/12,477/12,596	TTG/TTG/ATA/TTG	TAA/TAA/TAA/TAG	36/18/16/17
*trnL1*	N	12,489/12,441/12,483/12,597	12,552/12,504/12,542/12,660			1/1/22/0
*rrnL*	N	12,553/12,505/12,533/12,694	13,842/13,790/13,759/13,878			0/0/−15/33
*trnV*	N	13,843/13,791/13,845/13,962	13,912/13,860/13,914/14,031			0/0/17/83
*rrnS*	N	13,913/13,861/13,914/14,033	14,649/14,587/14,538/14,639			0/0/−1/1
*CR*		14,650/14,588/14,695/14,640	16,230/16,222/16,183/15,778			0/0/0/0

Note: The order of these four species in the table is as follows: *C. miliaris*, *L. cupreosplendens*, *S. insidious* and *P. rubroaureus*. ? represents ‘not determined’.

**Table 2:** Summarized mitogenomic characteristics of the four buprestid species investigated in this study.

Species	PCGs			rRNAs			tRNAs			A + T-Rich Region		
	Size (bp)	A + T (%)	A + T Skew	Size (bp)	A + T (%)	A + T Skew	Size (bp)	A + T (%)	A + T Skew	Size (bp)	A + T (%)	A + T Skew
*C. miliaris*	11,150	71.93	−0.15	2013	76.66	−0.03	1438	74.40	0.02	1635	78.58	−0.01
*L. cupreosplendens*	11,159	66.16	−0.16	2027	73.31	−0.07	1450	73.75	0.02	1581	76.58	0.03
*S.* *i* *nsidiosa*	11,034	71.46	−0.14	2076	76.74	−0.09	1452	73.77	0.01	1488	82.00	0.07
*P. rubroaureus*	11,140	67.92	−0.12	1790	72.18	−0.16	1410	70.85	0.03	1138	75.66	0.24

## Data Availability

The new sequences of complete mitogenomes can be available in NCBI (PV330099, PV330528, PV391144, and PX370042).

## Supplementary Materials

The following supporting information can be downloaded at: https://www.mdpi.com/article/10.3390/biom15111531/s1, Table S1: Information on the mitogenomes of Buprestidae and outgroup taxa used for phylogeny; Figure S1: The mitogenome maps of *Capnodis maliaris* (A), *Lamprodila cupreosplendens* (B), *Sphenoptera insidiosa* (C) and *Philocteanus rubroaureus* (D); Figure S2: The secondary cloverleaf structure for the tRNAs of *Capnodis maliaris*; Figure S3: The secondary cloverleaf structure for the tRNAs of *Lamprodila cupreosplendens*; Figure S4: The secondary cloverleaf structure for the tRNAs of *Sphenoptera insidiosa*; Figure S5: The secondary cloverleaf structure for the tRNAs of *Philocteanus rubroaureus*.
