# Supplementary material for: Mitochondrial Genome Features and Phylogenetic Analyses of Four Chrysochroinae Species (Coleoptera: Buprestidae)
Source: Biomolecules. 2025 Oct 30;15(11):1531. doi: 10.3390/biom15111531 (PMC12650119; doi:10.3390/biom15111531)
Supplement: Supplementary file 1 [file biomolecules-15-01531-s001.zip › biomolecules-3932011-supplementary.pdf]

Table S1. Information on the mitogenomes of Buprestidae and outgroup taxa used for phylogeny.

Taxa	Accession No.	Genome Size (bp)	A + T%	AT-Skew
Agrilus sichuanus Jendek, 2011	OK189519	16,521	71.73	0.12
Agrilus mali Matsumura, 1924	MN894890	16,204	74.46	0.08
Agrilus planipennis Fairmaire, 1888	KT363854	15,942	71.9	0.12
Agrilus discalis Saunders, 1873	ON644870	15,784	74.59	0.11
Coraebus diminutus Gebhardt, 1928	OK189521	15,499	68.42	0.12
Coraebus cloueti Théry, 1895	OK189520	15,514	69.27	0.11
Coraebus cavifrons Descarpentries & Villier, 1967	MK913589	15,686	69.79	0.12
Meliboeus sinae Obenberger, 1935	OK189522	16,108	72.42	0.11
Sambus femoralis Kerremans, 1892	OK349489	15,367	73.23	0.12
Sambus kanssuensis Ganglbauer, 1890	OQ784265	15,411	72.4	0.1
Habroloma sp.	OQ784266	16,273	73.99	0.11
Endelus continentalis Obenberger, 1944	OL702762	16,246	75.6	0.13
Cantonius szechuanensis Obenberger, 1958	OQ784264	15,927	73.09	0.11
Trachys auricollis Saunder, 1873	MH638286	16,429	71.05	0.1
Trachys troglodytiformis Obenberger, 1918	KX087357	16,316	74.62	0.1
Trachys variolaris Saunders, 1873	MN178497	16,771	72.11	0.11
Catoxantha luodiana (Yang & Xie, 1993)	PP211020	15,594	68.68	0.12
Anthaxia chinensis Kerremans, 1989	MW929326	15,881	73.61	0.09
Coomaniella dentata Song, 2021	OL694144	16,179	76.59	0.01
Coomaniella copipes Jendek & Pham, 2013	OL694145	16,196	74.47	0.03
Melanophila acuminata (De Geer, 1774)	MW287594	15,853	75.66	0.02
Nipponobuprestis guangxiensis Peng, 1995	PP133641	15,775	65.33	0.14
Chalcophora japonica (Gory, 1840)	OP388437	15,759	67.97	0.13
Chrysochroa fulgidissima (Schönherr, 1817)	EU826485	15,592	69.92	0.15
Dicerca corrugata Fairmaire, 1902	OL753086	16,276	71.76	0.09
Chrysochroa opulenta (Gory, 1832)	PP211021	15,587	67.16	0.16
Acmaeodera sp.	FJ613420	16,217	68.41	0.11
Ptosima chinensis Marseul, 1867	OP388449	16,115	67	0.13
Julodis variolaris (Pallas, 1771)	OP390084	16,227	70.43	0.12
Dryops ernesti Gozis, 1886	KX035147	15,672	72.98	0.07
Heterocerus parallelus Gebler, 1830	KX087297	15,845	74.03	0.13
Agrilus adelphinus Kerremans, 1895	NC_071932	15,732	71.35	0.1
Agrilus zanthoxylumi Li,1989	NC_081980	16,320	74.7	0.08
Buprestis fairmairei Théry, 1910	PV339624	13,390	73.42	0.09

Chrysobothris shirakii Miwa & chûjô, 1935	PV339621	15,789	78.54	0.02
Chrysobothris violacea Kerremans, 1892	PV339622	15,961	79.29	0.02
Phaenops yin Kubáň & Bíly, 2009	PV339623	16,051	76.51	0.04
Capnodis miliaris (Klug, 1829)	PV330099	16,230	73.46	0.04
Lamprodila cupreosplendens (Kerremans, 1895)	PV330528	16,222	68.76	0.1
Sphenoptera insidiosa Mannerheim, 1852	PV391144	16,183	73.47	0.09
Philocteanus rubroaureus (De Geer, 1778)	PX370042	15,778	69.49	0.18
Pyrocoelia rufa (Olivier, 1886)	AF452048	17,739	77.41	0.11
Limonius minutus (Linnaeus, 1758)	KX087306	16,727	76.71	0.05

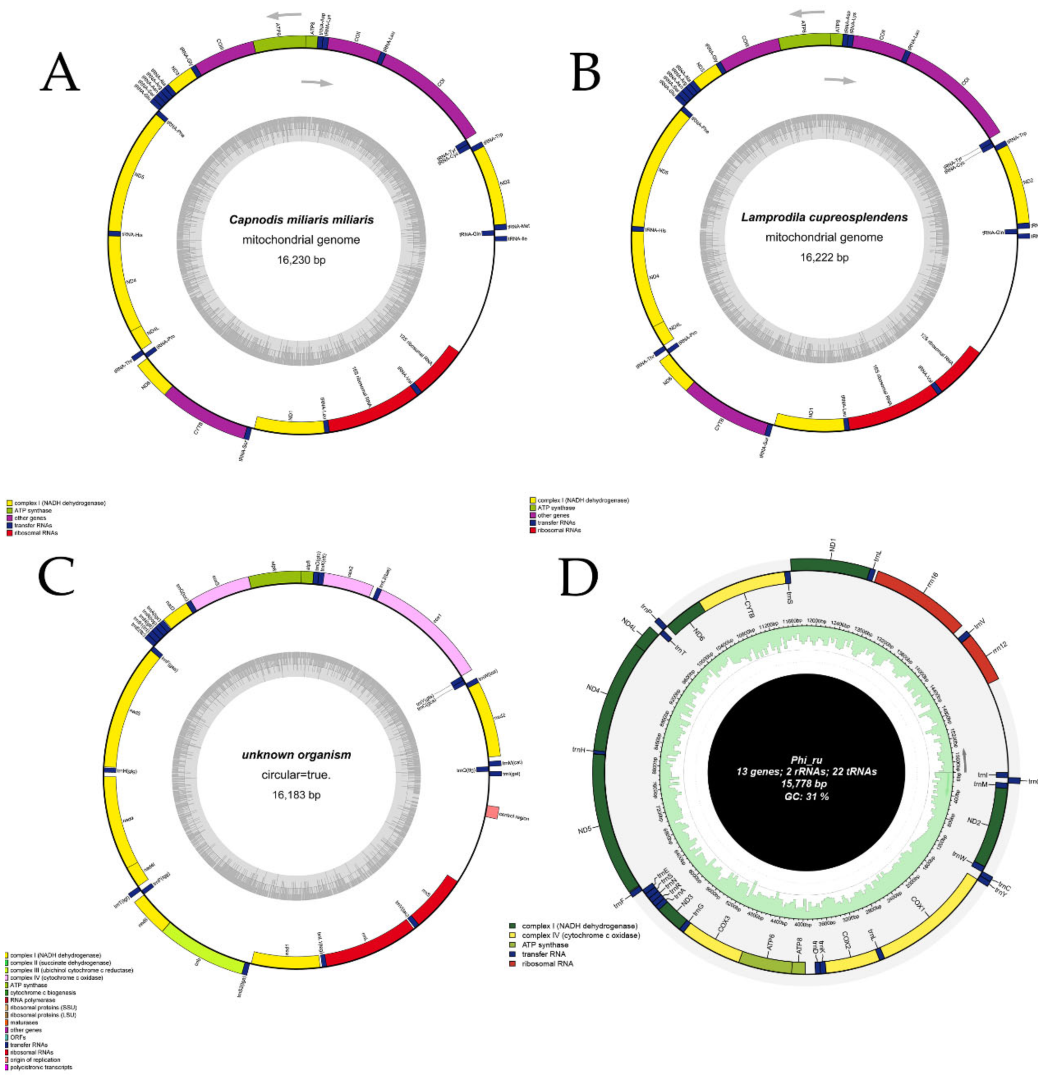

Figure S1. The mitogenome maps of *Capnodis miliaris* (**A**), *Lamprodila cupreosplendens* (**B**), *Sphenoptera insidiosa* (**C**), and *Philocteanus rubroaureus* (**D**).

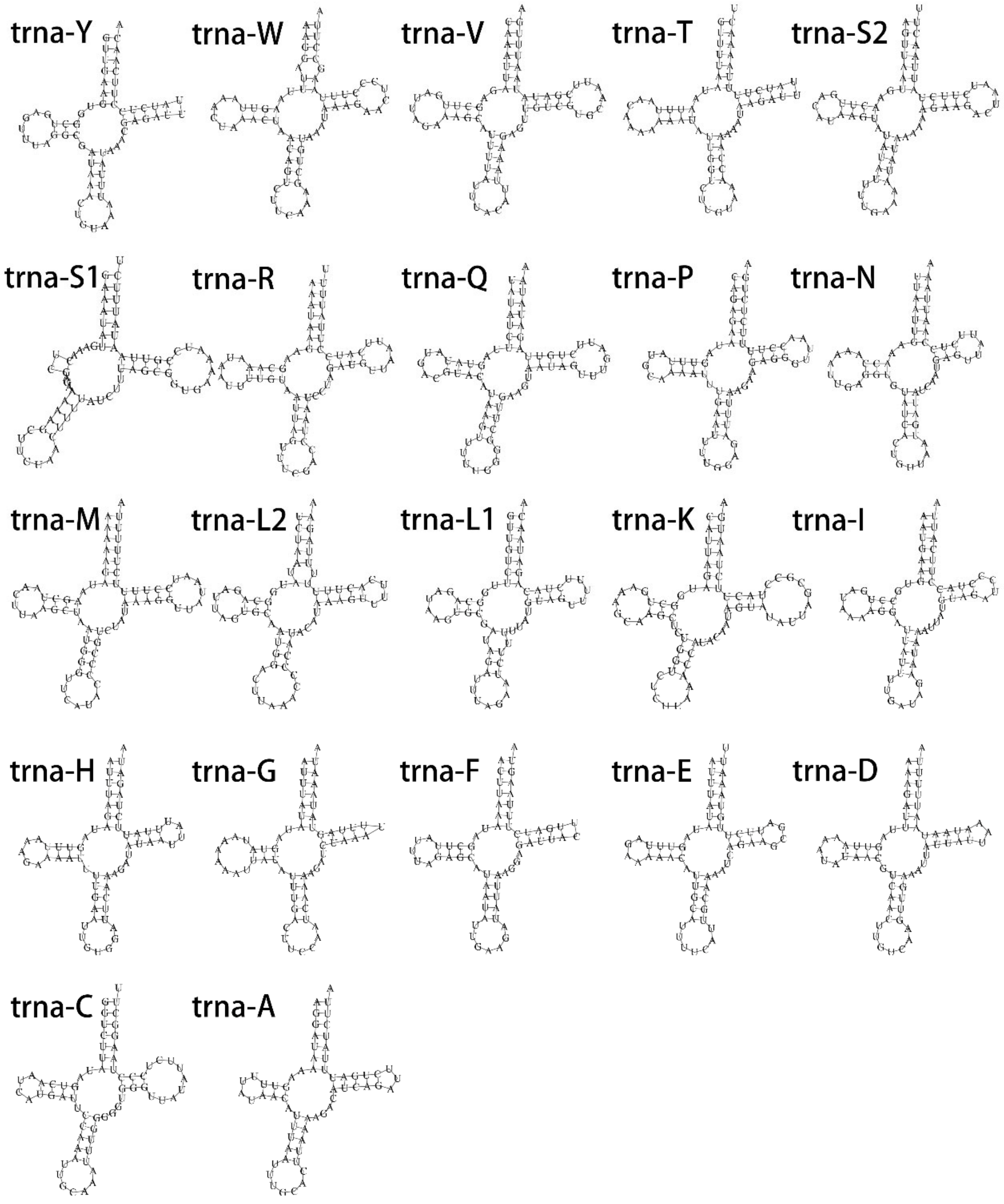

Figure S2. The secondary cloverleaf structure for the tRNAs of *Capnodis miliaris*.

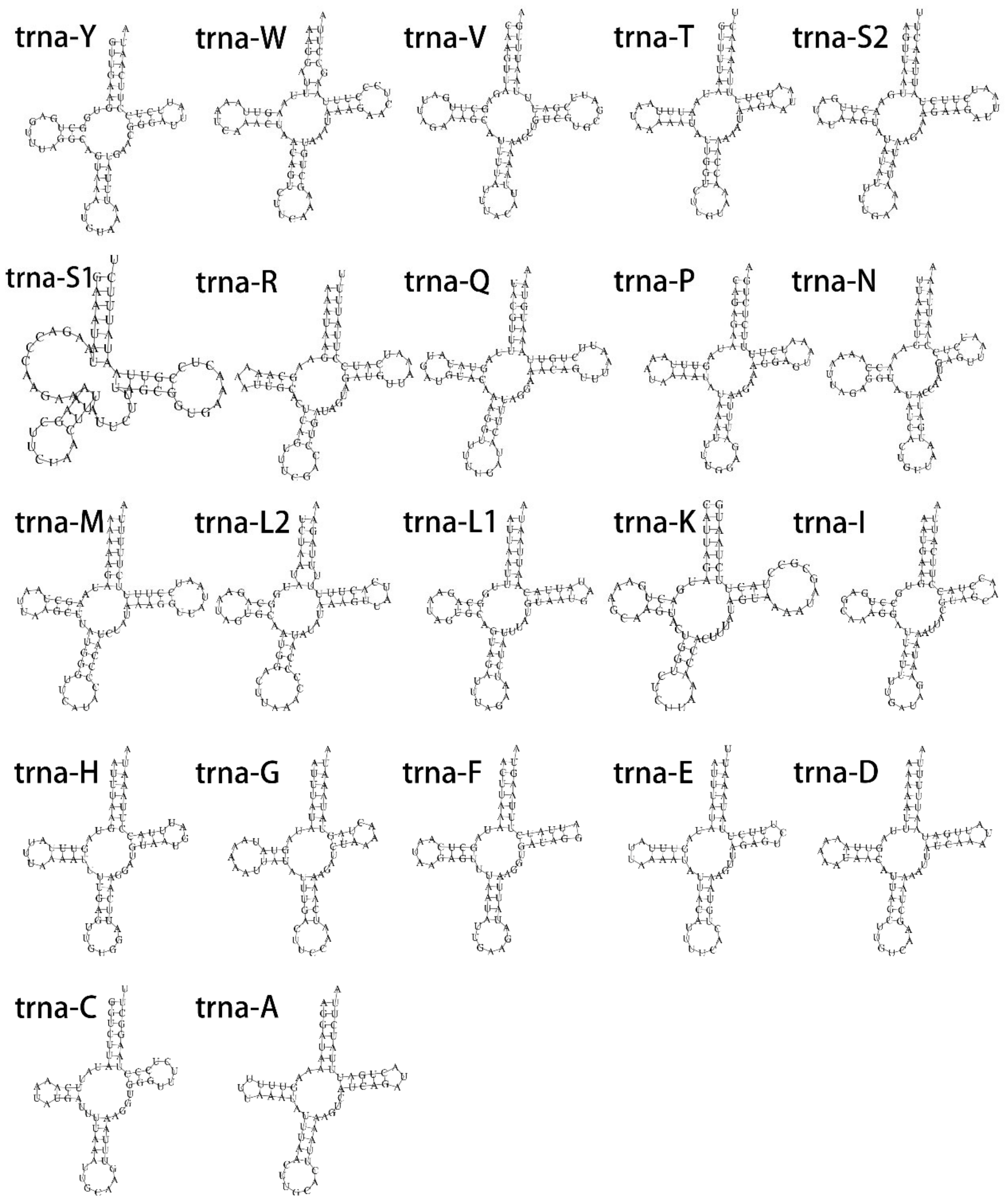

Figure S3. The secondary cloverleaf structure for the tRNAs of *Lamprodila cupreosplendens.*

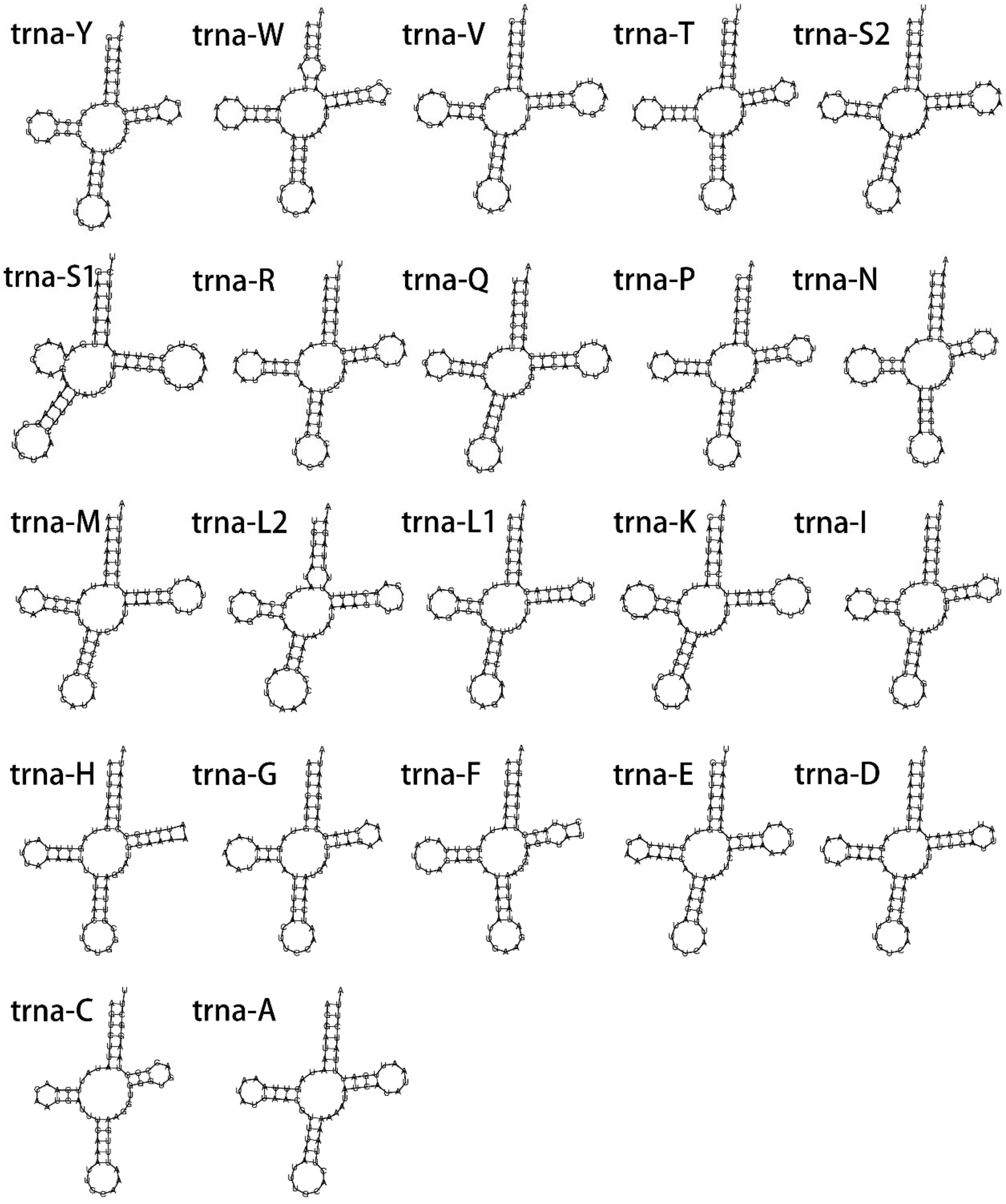

Figure S4. The secondary cloverleaf structure for the tRNAs of *Sphenoptera insidiosa*.

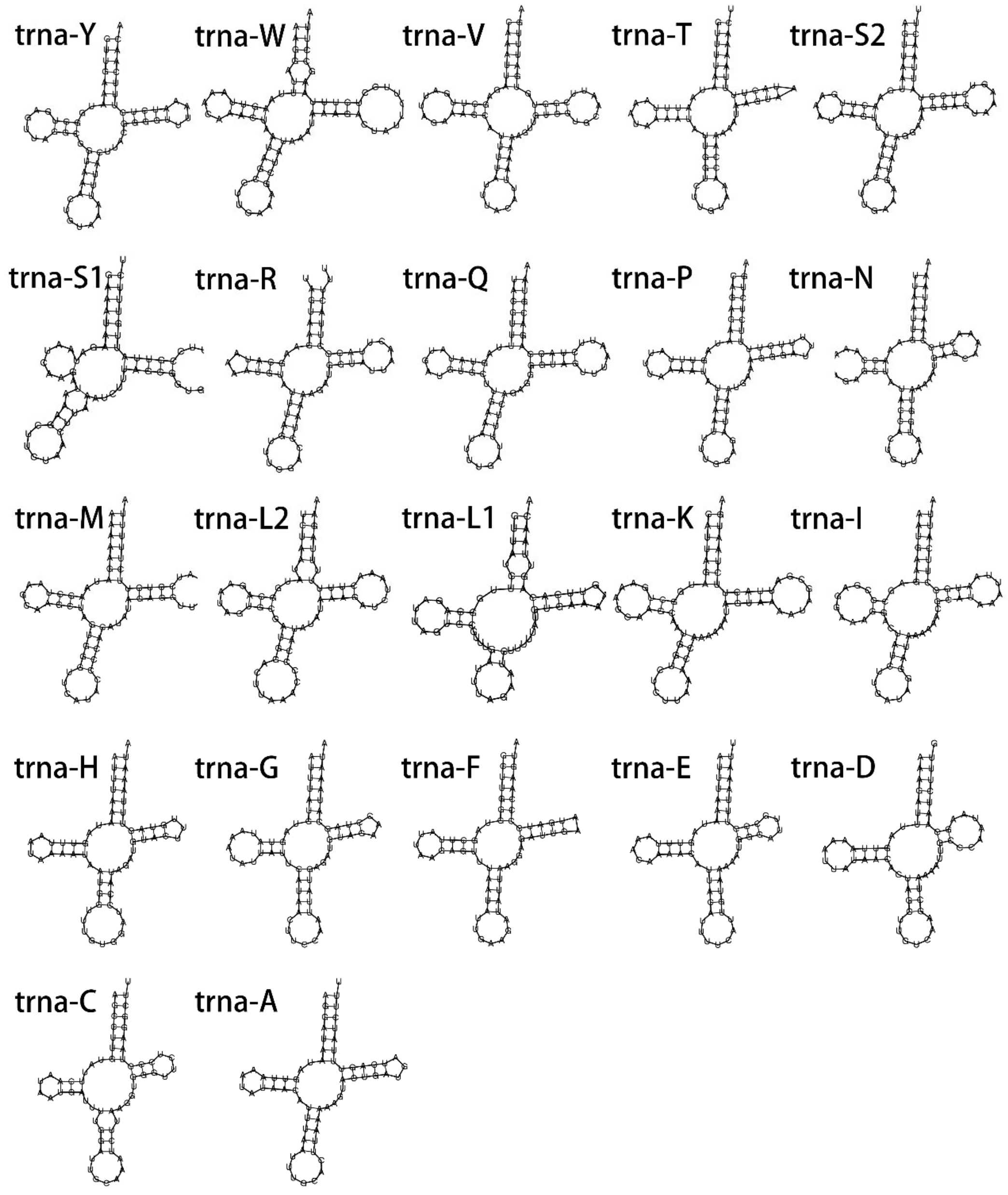

Figure S5. The secondary cloverleaf structure for the tRNAs of *Philocteanus rubroaureus*.
